# Peripheral Parenteral Nutrition and Personalized Nutritional Approach After Colorectal Resection Surgery: A Comprehensive Review of Current Evidence

**DOI:** 10.3390/medicina61081459

**Published:** 2025-08-14

**Authors:** Maximos Frountzas, Dimitrios Stefanoudakis, Evgenia Mela, Panagiotis Theodorou, George E. Theodoropoulos, Dimitrios Theodorou, Konstantinos G. Toutouzas

**Affiliations:** First Propaedeutic Department of Surgery, Hippocration General Hospital, School of Medicine, National and Kapodistrian University of Athens, 11527 Athens, Greece; stefanoudak@med.uoa.gr (D.S.); vemela@hotmail.com (E.M.); panptheodorou@gmail.com (P.T.); georgetheo@med.uoa.gr (G.E.T.); dtheodorou@med.uoa.gr (D.T.); tousur@med.uoa.gr (K.G.T.)

**Keywords:** colorectal surgery, postoperative nutrition, peripheral parenteral nutrition, nutritional status, inflammatory response, postoperative stress, postoperative recovery

## Abstract

Major surgical operations of the gastrointestinal tract, such as colorectal resections, lead to significant burden on the human body, which is expressed during the first postoperative hours with an intense inflammatory reaction and consumption of a large amount of energy, increasing patients’ nutritional requirements. Therefore, specific protocols have been implemented for the early initiation of oral feeding. However, not every patient could meet them due to old age and associated pathophysiological changes, the use of opioid drugs for the management of postoperative pain (which is associated with postoperative ileus or nausea), as well as open resections which might lead to gastrointestinal impairment during the first postoperative days. Therefore, a tailored nutritional approach after colorectal resections seems necessary under specific conditions. Parenteral nutrition could be part of this personalized treatment, as it might counterbalance the energy deficit occurring during the early postoperative period, which appears to be associated with adverse clinical outcomes. Nevertheless, the conventional way of administration through central venous lines is associated with significant complications. On the other hand, the alternative administration of parenteral nutrition through a peripheral venous catheter could avoid morbidity, maintaining patients’ energy balance even during the first postoperative hours. However, the efficacy of peripheral parenteral nutrition on the postoperative outcomes of patients undergoing colorectal resections needs to be investigated in prospective randomized trials. The aim of the present review is to present the current trends regarding administration of peripheral parenteral nutrition (PPN) after colorectal resections and highlight any potential correlations between PPN and postoperative inflammatory reaction, as well as short-term nutritional status.

## 1. Introduction: Postoperative Stress and Metabolic Disturbances

Major surgical operations, like colorectal resections, lead to traumatic injury for patients, causing the production of stress hormones and inflammatory mediators, which result in consumption of glycogen, proteins, and lipids, being part of the physiological response of healing [1]. That response to surgical stress could lead to increased catecholamine, cortisol, and glucagon levels, resulting in high insulin resistance during the early postoperative hours [2]. In addition, muscle protein degradation is increased due to low function of intracellular insulin [3]. This leads to loss of lean body mass, including diaphragmatic and postural muscles, as well as impaired mobility and respiratory capacity. During early postoperative period, there is also a shift from anabolic to catabolic state due to inhibition of the Insulin/Insulin-like Growth Factor 1 (IGF-1) signalling pathway at the level of Insulin Receptor Substrate 1 (IRS-1)/Phosphoinositide 3-Kinase (PI3K)/Protein Kinase B (Akt) [4,5]. Importantly, this shift could persist for some days, affecting wound healing, infection control, and functional recovery. Moreover, metabolic derangements are not limited to glucose metabolism. Amino acid oxidation increases due to IL-6-induced hepatic acute-phase response, while the upregulation hepatic urea cycle exacerbates nitrogen losses [6]. Increased mitochondrial reactive oxygen species (ROS) and altered redox states further contribute to local and systemic inflammation.

Notably, these physiologic reactions could affect postoperative outcomes of patients undergoing colorectal resection surgery. For instance, increased levels of postoperative glucose have been associated with several postoperative complications after colorectal surgery operations, while muscle degradation and functional impairment of such patients delay their recovery [7]. This major response to surgical injury is mediated by an intense inflammatory reaction, which is facilitated by pre-inflammatory cytokines, such as tumour necrosis factor-α (TNF-a), interleukin-1 (IL-1), IL-6, IL-8, IL-10, hormones (catecholamines, adrenocorticotropic hormone, cortisol, and glucagon), and chemokines [8,9]. Understanding the pathophysiology of surgical stress could help designing nutritional interventions to attenuate catabolism, support immune function, and promote recovery.

## 2. Perioperative Malnutrition

The impact of surgical stress on patients’ recovery could be precipitated by perioperative malnutrition. In fact, patients with severe underlying diseases, such as colorectal cancer, present malnutrition in 10–20% of cases at the time of surgery [10]. Perioperative malnutrition in such patients could be caused by gastrointestinal obstruction, malabsorption of nutritional elements throughout the gastrointestinal tract and preoperative chemo-radiotherapy in case of rectal cancer, which causes nausea and intestinal injury [11]. Furthermore, immunosenescence in elderly patients compounds malnutrition-related risks. Older adults often have reduced gastric acid production, altered zinc and vitamin D absorption, as well as age-related muscle loss (sarcopenia), all of which predispose to complications [12]. Opioid analgesics and anticholinergic medications further impair appetite and gastrointestinal transit. Moreover, perioperative malnutrition has been associated with decreased overall and disease-free survival in patients with gastrointestinal cancer [13]. Malnutrition not only impairs tissue repair and immune competence, but also disrupts gut barrier integrity, increasing the risk for bacterial translocation and systemic sepsis. In addition, malnutrition has been associated with increased rates of anastomotic leak, pneumonia, wound dehiscence, and delayed return of bowel function in surgical patients [14].

### 2.1. Malnutrition Screening Assessment

Under these circumstances, the European Society of Clinical Nutrition and Metabolism (ESPEN) recommends perioperative screening nutritional assessment in patients undergoing major gastrointestinal operations, identifying nutritional status as an important risk factor for postoperative clinical course [15]. Laboratory markers, including serum albumin, prealbumin, transferrin, and C-reactive protein could offer insight into both nutritional status and systemic inflammation [16]. In patients identified as malnourished or at nutritional risk, oral nutritional supplements (ONSs) are often indicated as a first-line, non-invasive intervention. These include energy-dense, protein-enriched formulas with added micronutrients, aimed at correcting deficits prior to surgery. A recent meta-analysis from Knight et al. has shown that preoperative ONSs can reduce all-cause postoperative complications and postoperative mortality [17]. In patients with obstructive or functional limitations to oral intake, high-protein liquid formulas or elemental diets may be used to bypass impaired digestive processes. The provision of targeted preoperative supplementation could attenuate the catabolic impact of surgery, modulate the inflammatory response, and reduce postoperative reliance on parenteral nutrition. Thus, comprehensive preoperative nutritional assessment not only identifies at-risk individuals, but also enables a proactive strategy to bridge nutritional gaps.

### 2.2. Prehabilitation and Exercise

Exercise-based prehabilitation represents a proactive strategy to optimize patients’ physiological reserves prior to colorectal surgery, particularly in those at risk for malnutrition or functional decline. Prehabilitation programmes typically combine aerobic conditioning, resistance training and respiratory muscle exercises initiated 2–4 weeks before the scheduled operation. These interventions aim to increase muscle mass, enhance cardiorespiratory fitness, and improve insulin sensitivity—physiological parameters that are often impaired in malnourished or elderly patients [18]. When combined with nutritional support, prehabilitation promotes anabolic adaptation, improves nitrogen balance, and attenuates the inflammatory response to surgical trauma. Functional assessments such as grip strength, gait speed, and six-minute walk distance are used to tailor exercise intensity and monitor progress [19]. A recent randomized controlled trial demonstrated the benefit of multimodal prehabilitation in terms of severe complications, medical complications, and functional capacity following colorectal surgery [20]. Integration of prehabilitation aligns with the principle of enhancing resilience through patient-centred interventions. Therefore, alongside preoperative nutritional assessment and oral supplementation, structured physical training could improve surgical preparedness, mitigate perioperative risk, and promote a faster and safer recovery following colorectal resection surgery.

### 2.3. Enhanced Recovery After Surgery (ERAS) Protocol

Ultimately, integration of nutritional screening, as well as food and physical supplementation into routine surgical planning fosters a personalized, preventive approach to perioperative care, aligning with the principles of the Enhanced Recovery After Surgery (ERAS) protocol and supporting improved functional recovery. ERAS protocol, which suggests starting of oral feeding during the first 24 h after colorectal resections, has further highlighted the importance of timely return to preoperative nutritional habits [21]. In most institutions, oral feeding starts on postoperative day 0, regardless of signs of recovered bowel function, such as bowel movements or flatus. This strategy leads to lower 30-day morbidity, shorter length of stay, and reduced hospital costs, whereas there is no difference in serious adverse events compared to the conventional feeding pathway after colorectal surgery [22]. In addition, the implementation of ERAS guidelines has been associated with better tissue healing, shorter length of postoperative hospital stay, decreased readmission rates, and lower hospital costs [23].

However, starting oral feeding early is not feasible for a large number of patients undergoing colorectal resection surgery, especially for the elderly and patients undergoing open surgery. Past medical history and age-related pathophysiological alterations are the main factors that make the response to postoperative surgical stress even more difficult for the elderly [24]. Opioid administration for the management of postoperative pain has been associated with nausea, vomiting, decreased appetite, and delayed oral feeding [25]. Finally, postoperative ileus in patients undergoing open colorectal resections and lack of education about nutritional postoperative recovery might lead to delays in reaching postoperative nutritional goals [26]. This highlights the need for alternative or bridging strategies, such as peripheral parenteral nutrition (PPN), to address early nutritional deficits when ERAS fails.

## 3. The Role of Peripheral Parenteral Nutrition

In the context of perioperative malnutrition, parenteral nutrition could improve postoperative nutritional status, especially in patients who cannot meet the ERAS guidelines after colorectal resection. Parenteral nutrition administration could lead to quick recovery from the early postoperative nutritional deficiencies, whereas it could prevent energy loss [27]. This is achieved through several biomolecular pathways. Parenteral nutrition activates protein translation and transcription, decreases autophagy and lysosome degradation of cellular products, while it enhances immune response by activating lymphocyte production [28]. However, the conventional administration of parenteral nutrition by using a central vein catheter has been associated with several complications, such as pneumothorax, catheter-related bloodstream infections, and great vessel thrombotic events [29]. Alternatively, parenteral nutrition could also be administered by a peripheral vein catheter, reaching postoperative nutritional goals and decreasing response to surgical stress during the first three postoperative days [30]. PPN represents a milder, short-term solution to address the early postoperative nutritional gap in patients unable to meet their caloric needs via the enteral route. Unlike total parenteral nutrition (TPN), which requires central venous access and carries higher risks of infection and thrombosis, PPN is administered via peripheral veins and is suited for partial supplementation over 3–7 days. PPN regimens contain lipids, glucose, amino acids, and oleic acid, which enhance immune response and decrease oxidative stress, as well as inflammatory reaction after major surgery [31]. The goal is to maintain nitrogen balance, preserve lean mass, and prevent metabolic deterioration during the acute phase of surgical recovery. Clinical trials have demonstrated that PPN improves protein kinetics and nitrogen balance, despite the presence of ongoing low-grade inflammation [32].

### 3.1. PPN in Colorectal Surgery

A randomized controlled trial has shown the potential benefit of PPN administration during the early postoperative period in 20 patients who had undergone colorectal resection surgery. The infusion of 2 litres PPN from the 1st to the 6th postoperative day, without concerning oral feeding level, was related to improved nitrogen balance during the first five postoperative days compared to saline administration, highlighting an important beneficiary effect on postoperative protein loss [33]. In addition, a retrospective study of 40 malnourished patients who underwent colorectal resection surgery, presented that PPN infusion during the first five postoperative days in combination to oral feeding led to higher postoperative albumin levels, earlier mobilization and shorter postoperative hospitalization length [34]. Finally, a recent randomized trial of 158 patients who underwent colorectal resection surgery due to cancer showed that early PPN administration was associated with lower postoperative morbidity and less severe postoperative complications. Interestingly, PPN infusion led to prevention of postoperative complications in 28% of patients who did not meet ERAS guidelines at the first postoperative day (Table 1) [35].

Importantly, PPN is not intended to replace enteral nutrition, but to complement or bridge until full oral intake is achieved. This is especially critical during the immediate postoperative period, when the gastrointestinal tract may be non-functional due to ileus, adhesions, or surgical handling. By supporting early protein and energy needs, PPN reduces muscle catabolism and may improve immune competence. Emerging strategies include personalized PPN regimens, where macronutrient ratios and infusion rates are tailored to age, renal function, surgical extent and inflammatory burden. Advances in emulsion technology (e.g., SMOFlipid, Fresenius Kabi, Bad Homburg, Germany) and fish oil-based emulsions) also offer new possibilities for immunomodulatory PPN [36].

### 3.2. Practical Aspects Regarding PPN Use

The satisfactory implementation of PPN in clinical setting requires a coordinated, multidisciplinary approach to ensure safety, efficacy, and timely delivery. Nowadays, most hospitals are supplied with pre-formulated PPN solutions containing specific amounts of nutrients. However, dietician teams usually make amendments to the pre-formulated PPN solutions according to patients’ needs [37]. Pharmacy departments are responsible for checking these amendments and ensuring correct formulation for each patient. Once prepared, solutions must be clearly labelled with composition, date of preparation, expiry, and patient identifiers, and stored under appropriate conditions until administration. Nursing staff play a pivotal role in PPN administration, monitoring, and line care. Peripheral venous access is usually established using a dedicated cannula (typically 18 G or 20 G) to minimize irritation and reduce the risk of phlebitis. Infusion rates should not exceed 80–100 mL/h to avoid vein inflammation, with the duration of use limited to 5–7 days [38]. Regular flushing of the line with normal saline is recommended to maintain patency. Infusion protocols often involve continuous administration over 16–24 h, tailored to patient tolerance and metabolic needs [39].

### 3.3. Adverse Effects of PPN

However, PPN is not without risks. Prolonged use can cause phlebitis, fluid overload, or electrolyte disturbances, and requires daily biochemical monitoring [40]. A notable limitation of PPN lies in its formulation constraints, particularly regarding lipid content. Due to the risk of thrombophlebitis and the limited osmolarity tolerance of peripheral veins, PPN solutions must remain below certain osmolar thresholds—typically around 800–900 mOsm/L [41]. As a result, lipid emulsions, which are energy-dense but contribute significantly to osmolarity, are often excluded or limited in PPN formulations [42]. This restricts the caloric density of the solution and may render PPN insufficient for meeting full energy requirements, especially in patients with high metabolic demands. Appropriate patient selection, short-term use, and multidisciplinary oversight are crucial for safe administration.

## 4. Nutritional Modulation in Colorectal Surgery

PPN could be used in combination with tailored medical treatments, which personalize clinical interventions based on the unique biological characteristics and needs of individual patients. In colorectal surgery, perioperative nutritional strategies—including the use of PPN—can be integrated into personalized care models, aiming to modulate stress responses, enhance recovery and reduce complications based on patient-specific profiles. Patients undergoing colorectal surgery exhibit heterogeneous responses to surgical stress due to genetic, metabolic, immunologic, and microbiome-related differences. For instance, variations in the expression of pro-inflammatory cytokines, such as IL-6 and TNF-α, or polymorphisms in genes encoding stress-related proteins, can significantly affect the magnitude and duration of the postoperative inflammatory response [43]. Tailoring nutritional support to accommodate these differences might affect patient outcomes and minimize the adverse effects of under- or over-nutrition during the recovery phase.

Emerging technologies, like metabolomics and proteomics, could offer real-time insights into a patient’s nutritional and metabolic state. By analyzing plasma metabolites and protein signatures before and after surgery, clinicians can predict catabolic stress, nutrient deficiencies, and immune activation. These tools may eventually support algorithms that optimize PPN formulation and timing in a truly individualized fashion [6]. Moreover, machine learning approaches are increasingly being used to predict postoperative nutritional needs. Data-driven models incorporating preoperative nutritional scores, comorbidity indices, intraoperative metrics, and early postoperative markers can stratify patients based on their likelihood of achieving ERAS nutritional goals [44]. In high-risk patients, early PPN initiation could then be employed proactively rather than reactively.

## 5. Health Economics of PPN

### 5.1. PPN and Healthcare Cost Reduction

Increased hospital cost could be considered as a potential drawback in PPN utilization. However, it seems to balance overall costs, particularly for patients undergoing major abdominal surgeries, like colorectal resections. These patients often experience delayed return of gastrointestinal function and failure to meet early oral intake goals that could lead to prolonged hospitalization and higher complication rates [45]. Direct costs associated with PPN include nutrient solutions, intravenous equipment, nursing time for line care, and monitoring and laboratory assessments for safety. On the other hand, early PPN might prevent postoperative complications, such as infections, dehiscence, or anastomotic leaks, which might reduce indirect costs associated with extended inpatient stays, reoperations, and ICU admissions [32]. These avoided costs can be substantial. Reducing postoperative complications after colorectal surgery could lead to healthcare cost reduction. A retrospective study in Singapore indicated a significant cost reduction by USD 0.31 million after the implementation of a surgical quality improvement programme which led to significant reduction in postoperative length of stay [46].

### 5.2. Bespoke Use of PPN

Furthermore, resource optimization through risk stratification enables targeted PPN use in malnourished or frail patients. This selective approach aligns with the concept of value-based care—maximizing benefit while minimizing cost. Predictive tools such as the Patient-Generated Subjective Global Assessment (PG-SGA), Global Leadership Initiative on Malnutrition (GLIM), Geriatric Nutritional Risk Index (GNRI), and Controlling Nutritional Status (CONUT), as well as machine learning-based risk calculators can help identify which patients will most likely benefit from early nutritional support [47]. From a broader system perspective, incorporating PPN into perioperative protocols may improve quality metrics, such as 30-day readmission rates, surgical site infection rates, and postoperative morbidity indices—factors that are increasingly linked to hospital reimbursement and accreditation status in many healthcare systems. Hence, health economic assessments must account not just for cost-savings, but also for value enhancement and healthcare service quality in outcome-driven care models [48].

## 6. Gut Microbiota and Perioperative Nutrition

### 6.1. Malnutrition and Dysbiosis

Outcome-driven policies often require measurable patient factors. Gut microbiota could be one of them. The interplay between the gut microbiota and host physiology might affect the recovery process after colorectal surgery. Surgical trauma, anesthesia, opioid use, and antibiotics could lead to microbial dysbiosis, which describes a state of reduced diversity, lower abundance of beneficial taxa, and overgrowth of pro-inflammatory strains of intestinal microbial flora [49]. This altered microbial landscape contributes to local inflammation, increased intestinal permeability, and translocation of endotoxins into systemic circulation [50]. Although PPN bypasses the gut, its role in preserving host–microbiota homeostasis should not be underestimated. Nutrient support reduces systemic stress and immunosuppression, indirectly supporting mucosal integrity [51]. Moreover, maintaining appropriate energy intake via PPN reduces the reliance on gluconeogenesis from muscle breakdown, thereby preserving amino acid pools necessary for gut barrier regeneration [52].

### 6.2. Nutritional Enhancement and Microbiome

Recent studies have explored the concept of microbiota-responsive nutrition where perioperative interventions are adapted based on microbiota signatures. For instance, metagenomic analysis of fecal samples has shown that patients with low levels of *Faecalibacterium prausnitzii* and *Bifidobacterium longum* are at higher risk for prolonged ileus and surgical site infections [53]. These patients may benefit from early PPN combined with microbiota-targeted therapies such as prebiotics (e.g., inulin), symbiotics, or selective digestive decontamination [53]. Moreover, gut–brain axis modulation has emerged as a novel mechanism linking microbiota alterations to postoperative pain perception, anxiety, and even delirium [54]. PPN might downstream these neurologic outcomes by attenuating the systemic inflammatory burden. In future practice, microbiota assessments may become a routine part of preoperative screening, guiding both the necessity and composition of PPN as part of an integrative nutritional plan.

## 7. Immunonutrition and PPN

### 7.1. Immunonutrition in Gastrointestinal Surgery

Nutritional interventions could also target specific regenerative human body systems, such as immunity system. In colorectal surgery, where the risk of infectious and inflammatory complications remains significant, immunonutrient-enriched PPN could emerge as an adjunct to standard nutritional support, as indicated by similar applications in gastric cancer surgery [55]. Key immunonutrients include

- Glutamine, which serves as a primary fuel for enterocytes and immune cells, enhances heat shock protein expression and reduces oxidative stress [56].
- Arginine, which promotes nitric oxide synthesis, enhances T-cell function and improves wound healing [57].
- Omega-3 fatty acids, which displace arachidonic acid in cell membranes and reduce pro-inflammatory eicosanoids and cytokine release [58].
- Nucleotides and selenium, which support cellular replication and antioxidant defence, respectively [59].

Retrospective studies in gastrointestinal surgical populations have demonstrated that perioperative immunonutrition—often as part of enteral regimens—reduces infectious complications by 25–30% and shortens hospital stay by 1–2 days [60]. However, evidence for immunonutrient use via PPN is still emerging [61]. Interestingly, the most recent meta-analysis about intravenous omega-3 lipid emulsion demonstrated reduction in systemic IL-6 and CRP levels postoperatively, but these differences failed to reach statistical significance [62].

### 7.2. Pharmaconutrition and PPN

Apart from immunonutrition, pharmaconutrition could play a role in tailored nutritional formulations. Patients with hyperinflammatory phenotypes may benefit from omega-3-rich PPN, while those with compromised antioxidant capacity (e.g., elderly or cancer patients) may require selenium or vitamin C supplementation [63]. Advanced monitoring—via dynamic cytokine panels or real-time metabolite analysis—will be essential to personalize dosing and duration. Additionally, incorporating these agents without destabilizing the PPN emulsion requires careful pharmaceutical planning and standardized regimen formula [64]. Regulatory bodies and professional societies, including ESPEN, currently recommend further research before routine use of immunonutrient-enriched PPN. However, with the growing interest in immune-nutritional synergy, this field is poised to become a major pillar of precision surgical care.

## 8. Future Research

### 8.1. Study Population and Intervention

Building the theoretical basis is very rare to change clinical practice. Well-designed interventions usually are required to improve clinical decision-making. The investigation of potential PPN efficacy on postoperative surgical stress and short-term nutritional status after colorectal resections requires meticulous future studies’ designing and multifactorial assessments. Therefore, such upcoming studies should prospectively enrol patients who will undergo colorectal operations at high-volume departments of surgery. A power analysis will be necessary before enrollment. A minimum of 250 patients should be included in such studies to reach statistically significant differences in the expected outcomes [65]. The inclusion criteria of future studies should be the following: (i) colorectal resection procedure; (ii) open, laparoscopic, or robotic procedures; (iii) primary anastomosis, defunctioning stoma or end stoma; (iv) elective or emergent operations; (v) small bowel resection combined with colorectal resection; vi) age ≥ 18 years old; (vii) informed consent for participation in this study. On the other hand, exclusion criteria will be the following: (i) small bowel resection only; (ii) defunctioning or end stoma without colorectal resection; (iii) preoperative administration of parenteral nutrition; (iv) parenteral nutrition prolongation after 5th postoperative day; (v) beginning of parenteral nutrition administration after the 1st postoperative day; (vi) contraindication for parenteral nutrition administration (e.g., allergy); (vii) age < 18 years old; (viii) no informed consent for study participation. The methodological design of such clinical trials should be accessible and their protocols should be registered in public trial registries.

A prospective randomized study design would enhance potential comparisons regarding postoperative stress response and short-term nutritional status. Patients who fulfil the inclusion criteria should be randomized by a software in two groups according to the “block randomization method” [66]. In the first group of patients (intervention group), 2000 mL of PPN with electrolytes (20 mL 7.45% KCl, 10 mL 20% MgSO_4_ and 20 mL 8.7% Na_3_PO_4_) could be administered via a peripheral venous catheter with a rhythm of 80 cc/h from the time they would leave the operation room until the 5th postoperative day [61]. In the second group of patients (control group), 1000 mL of 10% glucose saline with electrolytes (20 mL 7.45% KCl, 10 mL 20% MgSO_4_ and 20 mL 8.7% Na_3_PO_4_) could be administered via a central or peripheral venous catheter with a rhythm of 80 cc/h for the same time interval. Additional electrolyte and fluid administration according to the laboratory examinations and postoperative resuscitation should not be calculated to the aforementioned quantities and should be given according to each patient’s postoperative needs. Moreover, during the intervention interval (5 first postoperative days), a thorough clinical monitoring should be provided including frequent inspection of the infusion site and vital signs (systolic blood pressure, heart rate, respiration rate, temperature, and oxygen saturation) every 6 h to achieve a timely recognition of potential side effects from PPN infusion, such as thrombophlebitis and refeeding syndrome [67].

### 8.2. Study Outcomes

A digital platform, such as RedCap^©^ or Castor^©^, should be utilized for data collection to ensure data confidentiality and easy editing. Patients’ demographics (gender, age, smoking status, Body Mass Index (BMI), and physical status according to the ASA—the American Society of Anesthesiologists) and their past medical history (ischemic heart disease, diabetes, arrhythmia, hypertension, and anticoagulants) should be reported. Furthermore, a screening nutritional assessment based on the MUST (Malnutrition Universal Screening Tool) would be conducted to stratify patients according to their preoperative malnutrition risk, and their preoperative albumin levels would be reported as well [68]. The definite diagnosis should be reported, as well as the specific type of operation conducted and other intraoperative parameters, such as technical aspects of the operation and transfusion of blood product. Postoperative follow-up of patients would be conducted in several stages. Firstly, serum biochemical parameters regarding electrolytes (sodium, potassium, calcium, and magnesium), blood urea nitrogen, serum creatinine, blood glucose, and liver function enzymes (bilirubin, serum glutamic–oxaloacetic transaminase (SGOT), serum glutamic pyruvic transaminase (SGPT)) should be reported. In addition, hematocrit, hemoglobin levels, and white blood count levels would be retrieved. Postoperative follow-up should include side effects of PPN infusion, like thrombophlebitis, local swelling due to extravasation and refeeding syndrome. Moreover, total postoperative complications in 30 and 90 days after surgery should be reported. The type of complications and their severity according to the Clavien–Dindo classification should also be noticed [69]. Length of postoperative stay and gastrointestinal function-related outcomes, such as postoperative day of nasogastric tube removal, postoperative time to first flatus, postoperative day of per os feeding start, and postoperative day of total per os feeding would be reported. Finally, the implementation of postoperative ERAS parameters, such as per os liquid administration in the first postoperative 24 h, early mobilization in the first postoperative 24 h, and absence of postoperative analgesic medication including opioids, would be screened [70].

**Figure 1:**
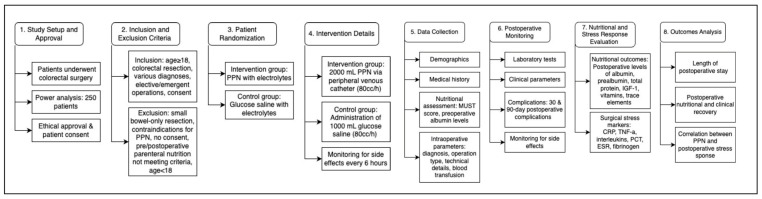
Flow diagram of the study from its setup until the outcome analysis.

The efficacy of PPN should be investigated by nutritional laboratory parameters, such as albumin, pro-albumin, transferrin, and total protein levels, as well as nitrogen balance calculation after surgery [71]. Moreover, several trace elements (vitamin D, vitamin B12, folate, zinc, and selenium) and insulin-like growth factor-1 (IGF-1) levels would be calculated [72,73,74]. On the other hand, the potential association of PPN with postoperative response to surgical stress could be investigated by serum tests including c-reactive protein (CRP), tumour necrosis factor-α (TNF-a), interleukin-1 (IL-1), IL-6, IL-8, IL-10, procalcitonin (PCT), fibrinogen, erythrocyte sedimentation rate (ESR), and tumour necrosis factor-alpha (TNF-α) levels [75,76,77,78,79,80]. A proposed study flow diagram is depicted in Figure 1.

## 9. Conclusions

**Figure 2:**
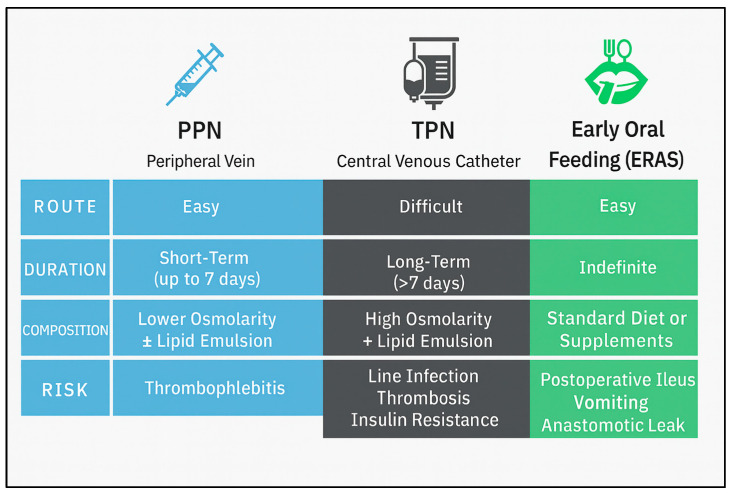
Comparison between different approached regarding nutritional replacement after colorectal resection surgery. PPN, peripheral parenteral nutrition; TPN, total parenteral nutrition; ERAS, enhanced recovery after surgery.

Conclusively, colorectal resection surgery causes a traumatic impact on human physiology with inflammatory reaction to surgical stress. The immediate postoperative period might play a crucial rule in counterbalancing metabolic needs and this could affect postoperative outcomes regarding morbidity and mortality [81]. The present review has demonstrated that the administration of PPN after colorectal resection surgery might counterbalance energy needs from the first postoperative hours (Figure 2). This could be a novel approach in handling postoperative surgical stress and nutritional defect after colorectal resection in patients who cannot rapidly return to their preoperative nutritional habits.

Despite the comprehensive analysis presented, this review has several limitations that must be acknowledged. Firstly, much of the evidence surrounding PPN in colorectal surgery derives from small-scale, single-centre studies, or retrospective analyses, which may limit generalizability and introduce selection bias (Table 1). The heterogeneity of patient populations, surgical techniques (open vs. minimally invasive), and nutritional protocols across studies further complicates the direct comparison of outcomes. Moreover, immunonutritional and microbiota-based interventions remain exploratory, with limited evidence supporting their integration into PPN protocols at this stage. Finally, while the review emphasizes the potential for personalized nutritional strategies, current clinical application is constrained by the absence of validated biomarkers and decision-making algorithms in routine practice.

Meticulously designed prospective randomized trials seem to be necessary to examine the potential utilization of PPN as an adjunct of ERAS protocol in cases when it cannot be implemented in the conventional way. The measurement of specific postoperative parameters which describe the effect of PPN on the nutritional status of patients undergoing colorectal resections and their response to surgical stress would allow the quantification of this effect, enabling specific prognostic models to be implemented in a wider clinical scheme.

## Figures and Tables

**Table 1:** Studies regarding role of parenteral peripheral nutrition after colorectal resection surgery. PPN; peripheral parenteral nutrition; POD, postoperative day.

Author; Year	No of Patients	Type of Study	Study Population	Intervention	Outcomes
Gys; 1990 [33]	20 patients	Randomized Controlled Trial	Patients undergoing colorectal resection	2L PPN daily from POD 1 to 6, regardless of oral intake	Improved nitrogen balance during first 5 postoperative days
Liu; 2013 [34]	40 patients	Retrospective Cohort Study	Malnourished patients post-colorectal resection	PPN + oral feeding during first 5 postoperative days	Higher postoperative albumin, earlier mobilization, shorter hospital stay
Sánchez-Guillén; 2021 [35]	158 patients	Randomized Controlled Trial	Colorectal cancer patients undergoing resection	Early PPN administration during immediate postoperative period	Lower postoperative morbidity, reduced severity of complications, 28% benefit in ERAS non-adherent patients

## Data Availability

Dataset available on request from the authors.
